# The role of the paraspinal muscles in the development of adolescent idiopathic scoliosis based on surface electromyography and radiographic analysis

**DOI:** 10.1186/s12891-024-07329-w

**Published:** 2024-04-03

**Authors:** Yinchuan He, Hongtao Dong, Ming Lei, Jianan Liu, Hongru Xie, Zepei Zhang, Jun Pang, Mengyue Jin, Jian Wang, Ziming Geng, Jing’an Zhang, Gang Li, Qilong Yang, Lin Meng, Jun Miao

**Affiliations:** 1 Graduate School, Tianjin Medical University, No. 22 Qixiangtai Road, Heping District, Tianjin, 300070 China; 2 Academy of Medical Engineering and Translational Medicine, Tianjin University, No. 92 Weijin Road, Nankai District, Tianjin, 300072 China; 3 Present Address: Department of Spine Surgery, Tianjin Hospital, Tianjin University, No.406, Jiefang South Road, Hexi District, Tianjin, 300210 China; 4 The Second Department of Radiology, Tianjin Hospital, Tianjin University, No.406 Jiefang South Road, Hexi District, Tianjin, 300210 China; 5 Department of Spine Surgery, Hebei Cangzhou Hospital of Integrated Traditional Chinese Medicine and Western Medicine, No.31 Huanghe Road, Cangzhou, 061001 China

**Keywords:** Surface electromyography, Paraspinal muscles, Idiopathic scoliosis, Cobb angle

## Abstract

**Background:**

Patients with idiopathic scoliosis commonly present with an imbalance of the paraspinal muscles. However, it is unclear whether this muscle imbalance is an underlying cause or a result of idiopathic scoliosis. This study aimed to investigate the role of paraspinal muscles in the development of idiopathic scoliosis based on surface electromyography (sEMG) and radiographic analyses.

**Methods:**

This was a single-center prospective study of 27 patients with single-curve idiopathic scoliosis. Posteroanterior whole-spine radiographs and sEMG activity of the erector spinae muscles were obtained for all patients in the habitual standing position (HSP), relaxed prone position (RPP), and prone extension position (PEP). The Cobb angle, symmetrical index (SI) of the sEMG activity (convex/concave), and correlation between the two factors were analyzed.

**Results:**

In the total cohort, the mean Cobb angle in the HSP was significantly greater than the mean Cobb angle in the RPP (RPP-Cobb) (*p* < 0.001), whereas the mean Cobb angle in the PEP (PEP-Cobb) did not differ from the RPP-Cobb. Thirteen patients had a PEP-Cobb that was significantly smaller than their RPP-Cobb (*p* = 0.007), while 14 patients had a PEP-Cobb that was significantly larger than their RPP-Cobb (*p* < 0.001). In the total cohort and two subgroups, the SI of sEMG activity at the apex vertebra (AVSI) in the PEP was significantly greater than 1, revealing significant asymmetry, and was also significantly larger than the AVSI in the RPP. In the RPP, the AVSI was close to 1 in the total cohort and two subgroups, revealing no significant asymmetry.

**Conclusion:**

The coronal Cobb angle and the SI of paraspinal muscle activity in AIS patients vary with posture changes. Asymmetrical sEMG activity of the paraspinal muscles may be not an inherent feature of AIS patients, but is evident in the challenging tasks. The potential significance of asymmetric paraspinal muscle activity need to be explored in further research.

## Background

Adolescent idiopathic scoliosis (AIS) is a three-dimensional spinal deformity that is defined as a lateral curvature of the spine in the coronal plane of > 10° in adolescents aged 10–18 years [1, 2]. The prevalence of idiopathic scoliosis in adolescents is 1−3% [3]. Among the patients with scoliosis, 80−85% are diagnosed with idiopathic scoliosis [4]. Despite many years of extensive research, the etiology of AIS remains unknown. Previous studies have indicated that patients with idiopathic scoliosis commonly present with an imbalance of the paraspinal muscles regarding the orientation, length, thickness, and fiber type composition [5–7]. The ligamentous spine cannot support longitudinal compressive forces, and an axial load of just 20 N can cause it to buckle [8]. As muscles are important for maintaining the stability of the upright spine, paraspinal muscle imbalance has long been considered a potential cause of AIS [9–14].

Surface electromyography (sEMG) is an objective and noninvasive technique that has been used to describe the electrical activity of the paraspinal muscles in AIS [15, 16]. Some studies have demonstrated that imbalanced paraspinal muscles cause scoliosis [10, 11, 17, 18]. In AIS, type I fibers are predominant on the convex side, while there are more type II fibers on the concave side. The presence of more type 1 fibers on the convex side may provide a sustained pull on the spine, resulting in scoliosis [11, 17]. Riddle and Roaf [10] reported that stronger electrical activity on the convex side produces rotation deformity in the first stage of scoliosis. However, EMG is not an accurate proxy for muscle force [19].Other studies have reported that paraspinal muscle imbalance is a secondary effect of spinal deformity [20–23]. The significantly lower proportion of type I fibers on the concave side may be a result of the disuse of the paraspinal muscles associated with spinal deformity [23]. Furthermore, increased muscle activity on the convex side may be a sign of muscle weakness [24], and an enhanced sEMG ratio at the lower end vertebra (LEV) is associated with the progression of scoliosis [16].

To date, it remains unclear whether paraspinal muscle imbalance is a cause or consequence of scoliosis. Previous studies have used sEMG to assess the paraspinal muscle performance of AIS patients during static and dynamic tasks [16, 25]. However, few studies have focused on the effect of the paraspinal muscle activities on the scoliotic Cobb angle in the coronal plane. It is reported that the erector spinae muscles are the main spine stabilizers [16, 26], whose activity contribute to the dorsal extension of the spine and also provide some lateral flexional force [27]. Unilateral activity gives the combined mechanical function of extension and lateral bending of the spine. Bilateral activity extends the spine and stabilizes the spine mainly in the lateral direction [28]. The erector spinae muscles are less activated in the relaxed prone position (RPP) [24] and more activated in the prone extension position (PEP) [29]. To investigate the role of paraspinal muscles in the development of idiopathic scoliosis, we performed sEMG and posteroanterior whole-spine radiography in three positions, including habitual standing position (HSP), RPP, and PEP. By evaluating the sEMG activity of the erector spinae muscles and the Cobb angles in response to three positions in AIS patients, we aimed to address the following four concerns: [1] Is paraspinal muscle activity asymmetric in three positions [2]? Whether the Cobb angle and the symmetry of paraspinal muscle activity change, when the position varies [3]. the relationship between the asymmetrical paraspinal muscle activity and the Cobb angle [4]. the potential significance of asymmetrical paraspinal muscle activity.

## Methods

Patients with adolescent idiopathic scoliosis received X-ray examinations and sEMG measurements in three positions, HSP, RPP and PEP. The Cobb angle and sEMG change were observed when patients changed positions from HSP to RPP and from RPP to PEP. Patients who tended to have smaller or the same Cobb angles from RPP to PEP were divided into group A, and who tended to have larger Cobb angles from RPP to PEP were divided into group B. The sEMG were analyzed for the correlation with Cobb angle in the three positions.

## Patients

Twenty-seven adolescents with idiopathic scoliosis were recruited from the Tianjin Hospital between October 2021 and August 2022 (Table 1). The inclusion criteria were: [1] diagnosis of single-curve AIS, including thoracic, lumbar, or thoracolumbar curves; [2] age between 10 and 18 years; and [3] no other pathological conditions. The exclusion criteria were: [1] a diagnosis of non-idiopathic scoliosis due to congenital, neuromuscular, or other connective tissue disease; [2] a history of spinal exercise, bracing, or surgical treatment.

**Table 1:** Demographic characteristics of the patients (mean ± SD)

	Total cohort (*n* = 27)	Group A (PEP-Cobb < RPP-Cobb) (*n* = 13)	Group B (PEP-Cobb > RPP-Cobb) (*n* = 14)	Statistics	p
Gender (f/m)	20/7	10/3	10/4	-	1.000 ^a^
Age (years)	14.30 ± 1.38	13.92 ± 1.44	14.64 ± 1.28	t= -1.376	0.181
Height (m)	1.63 (1.60, 1.66)^b^	1.63 (1.60, 1.65)^b^	1.67 ± 0.07	z = 0.659	0.519
Weight (kg)	51.56 ± 12.50	47.08 ± 10.59	55.71 ± 13.05	t= -1.876	0.072
BMI (kg/m^2^)	17.85 (16.33, 20.15)^b^	17.45 (16.16, 18.64)^b^	20.27 ± 4.56	z = 1.407	0.169
Disease duration (months)	12.0(4.5, 18.0)^b^	6.0 (2.0, 21.0)^b^	12.0 (5.63,15.0) ^b^	z = 0.642	0.550
HSP-Cobb (°)	30.59 ± 11.69	25.68 ± 8.64	35.14 ± 12.57	t= -2.261	0.033*
Curve type (T%)	37.0	38.5	35.7	-	1.000 ^a^
Risser sign	4.0(2.0, 4.0)^b^	4.0(4.0, 4.0)^b^	3.5(2.0, 4.0)^b^	z=-1.047	0.295

^a^Significance assessed by Fisher’s exact test; ^b^: values are presented as median (P25, P75); HSP-Cobb: Cobb angle in the habitual standing position; Group A (PEP-Cobb < RPP-Cobb): patients in whom the Cobb angle in the prone extension position (PEP-Cobb) is smaller than or equal to the Cobb angle in the relaxed prone position (RPP-Cobb); Group B (PEP-Cobb > RPP-Cobb): patients in whom the PEP-Cobb is greater than the RPP-Cobb; BMI: body mass index; *p < 0.05 for the comparison of group A with group B

The Medical Ethics Committee of the hospital approved the study, and informed consent was obtained from all patients and their parents.

## Radiographic assessment

**Fig. 1:**
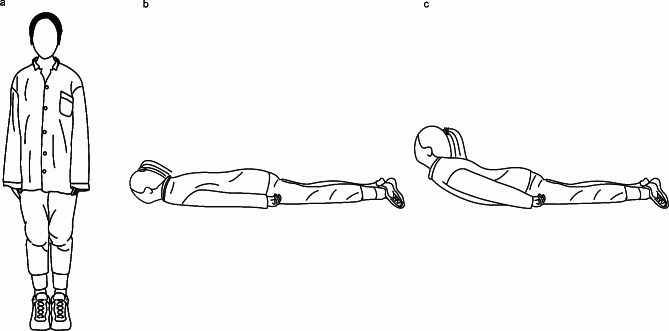
Test postures. **(a)** Habitual standing position. **(b)** Relaxed prone position. **(c)** Prone extension position

X-ray examinations were performed using a 500-mA digital radiography system (AXIOM Aristos VX Plus; Siemens Healthineers, Erlangen, Germany) with 75 kV in the posteroanterior position using an automatic exposure control system. Radiographs were obtained for all patients in the HSP, RPP, and PEP (Fig. 1). The upper end vertebra (UEV), apex vertebra (AV), and LEV of the scoliotic curve were determined on standing whole-spine radiographs. The Cobb angles in the HSP (HSP-Cobb), RPP (RPP-Cobb), and PEP (PEP-Cobb) were measured between the superior endplate of the UEV and the inferior endplate of the LEV by using Surgimap software (Nemaris Inc., New York, NY).

To measure the intra-examiner reproducibility and inter-examiner reliability of the radiographic parameters, two spinal surgeons independently performed the measurements twice with at least a 1-week interval between measurements. The intra-examiner reproducibility and inter-examiner reliability were examined using the intraclass correlation coefficient (ICC). According to the Fleiss guidelines [30], ICCs of less than ± 0.40 indicate poor reliability, ± 0.40–0.75 indicate fair or good reliability, and ± 0.75–1.00 indicate excellent reliability.

## Surface electromyographic measurements

The sEMG measurements were performed approximately 3 to 5 days after completing the X-ray examinations. A Noraxon EMG measurement system with wireless EMG sensors (Noraxon Inc., Scottsdale, AZ, USA) was used for the data collection at a sampling rate of 2000 Hz. In accordance with the Surface EMG for a Non-Invasive Assessment of Muscles standards [31], after skin preparation, the self-adhesive bipolar surface electrodes were placed 2.5 cm from the midline and fixed along the muscle fiber orientation on the bilateral erector spinae muscle (longissimus) bellies at the UEV, AV, and LEV levels [32]. Patients were asked to lift the trunk from a prone position in order to confirm that the electrodes were properly placed.

## Tasks and procedures

**Fig. 2:**
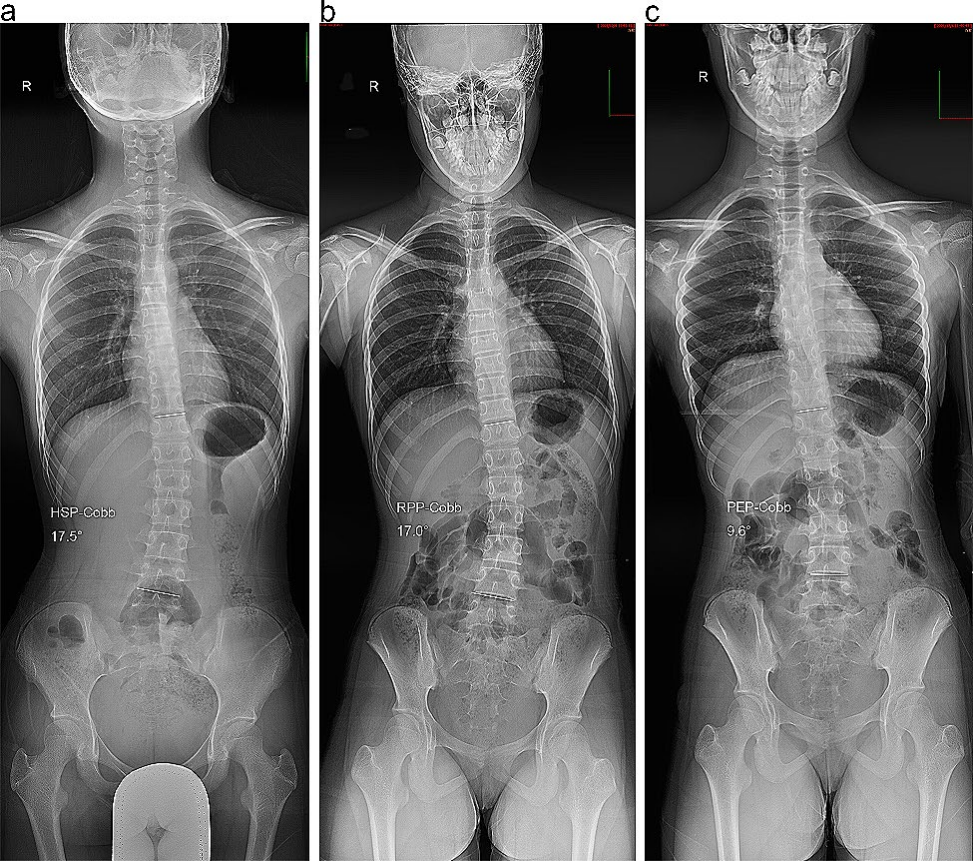
Cobb angles in group A. **(a)** Cobb angle in the habitual standing position (HSP-Cobb). **(b)** Cobb angle in the relaxed prone position (RPP-Cobb). **(c)** Cobb angle in the prone extension position (PEP-Cobb). The PEP-Cobb is smaller than the RPP-Cobb

**Fig. 3:**
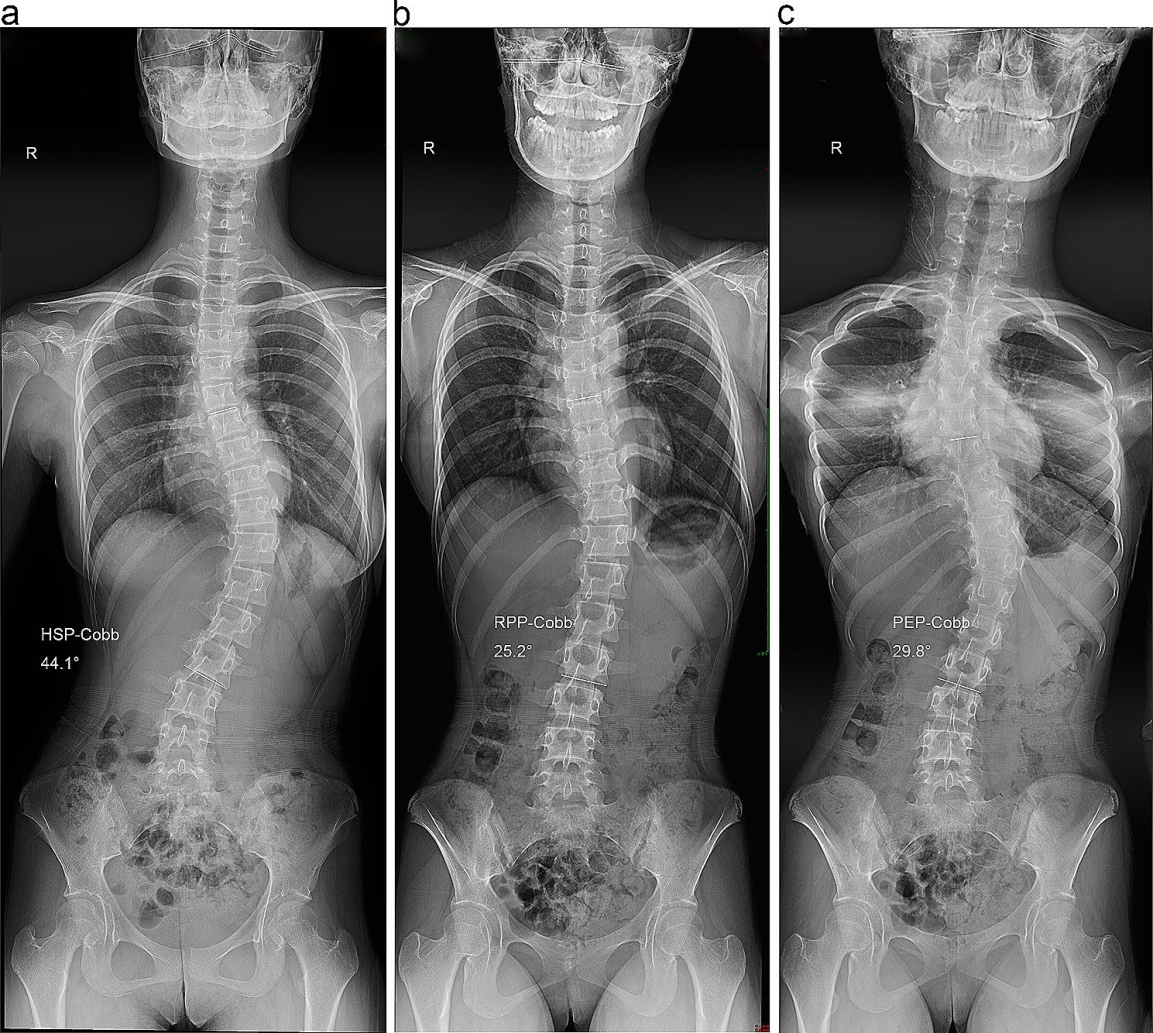
Cobb angles in group B. **(a)** Cobb angle in the habitual standing position (HSP-Cobb). **(b)** Cobb angle in the relaxed prone position (RPP-Cobb). **(c)** Cobb angle in the prone extension position (PEP-Cobb). The PEP-Cobb is larger than the RPP-Cobb

The test tasks were recorded with the patient in three postures: HSP, RPP, and PEP. Each task was recorded three times. Each recording lasted five seconds. Before recording, patients were given the following precise instructions [1]. For the HSP, the patients were asked to stand in the resting state (Fig. 1a); [2] for the RPP, the patients lay prone with their arms along the trunk, with the body in resting position and the muscles relaxed (Fig. 1b); [3] for the PEP, the patients raised their trunk off the table as much as possible and sustained a static position (Fig. 1c). To avoid fatigue, every patient had a 2-minute rest period between any two consecutive tests.

## Surface electromyography signal processing

The raw sEMG signals were processed using a Butterworth bandpass filter of 15–500 Hz and notch filter of 50 Hz to reduce the electrical interference from external sources. After rectification of the data, the root mean square (RMS) values were calculated with a 2-second time window. Raw sEMG data underwent repeatability testing using an ICC ranging from 0.825 to 0.945. The average RMS values of the three tests were subsequently analyzed. The symmetrical index (SI) of the paraspinal muscle activity was calculated as follows: SI = RMS (convex)/RMS (concave) [33]. A ratio of 1 indicates that the tested muscle pair has relatively symmetrical sEMG activity between the convex and concave sides. If the SI is larger than 1, the convex side of the muscle has relatively greater sEMG activity than the concave side. If the SI is less than 1, the convex side of the muscle has relatively lesser sEMG activity than the concave side.

## Statistical analysis

Statistical analyses were carried out using SPSS statistical package (version 25.0; SPSS, Chicago, IL, USA). The Shapiro-Wilk test was used to verify the normality of distributions. The nonparametric one-sample Wilcoxon signed rank test was performed to identify any asymmetry (deviation from the test value of 1) in sEMG activities. One-way repeated measure analysis of variance and the Friedman’s two-way analysis of variance by ranks were used to compare the Cobb angles and SI values in the three postures. A post hoc test with a Bonferroni correction was applied to determine the differences in the Cobb angle and SI between postures. The independent t-test and Mann-Whitney U test were used to compare parameters between subgroups. Fisher’s exact test was used to analyze subgroup frequencies in contingency tables. Statistical significance was set at *p* < 0.05.

## Results

## Radiographic results

**Fig. 4:**
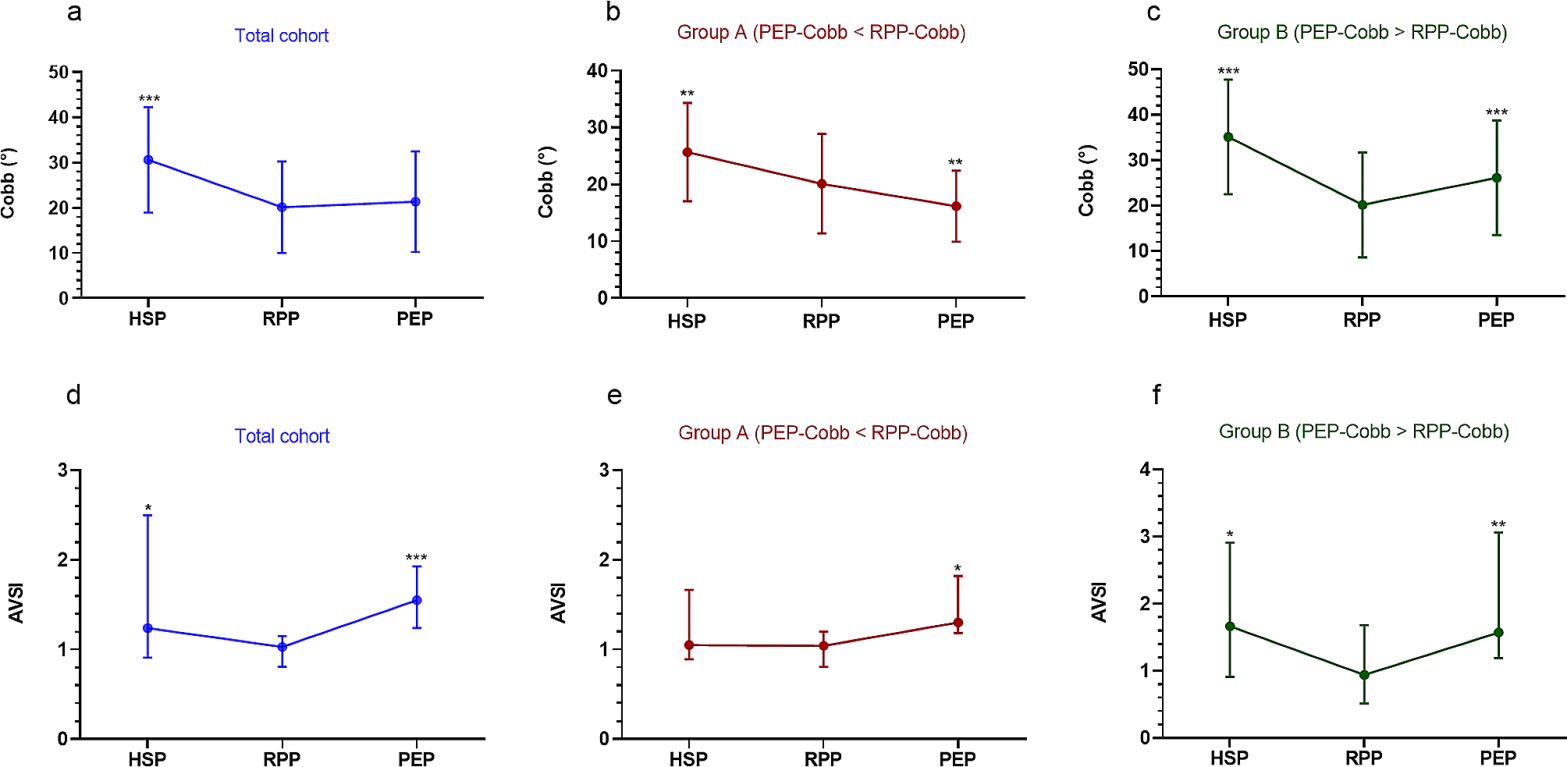
Changes in the Cobb angle and AVSI between postures. **(a)** In the total cohort, the Cobb angle is significantly greater in the habitual standing position (HSP) than the relaxed prone position (RPP), and the Cobb angle in the prone extension position (PEP) does not significantly differ from the Cobb angle in the RPP. **(b)** In group A (PEP-Cobb < RPP-Cobb), the Cobb angle is significantly greater in the HSP than the RPP, while the Cobb angle is significantly smaller in the PEP than the RPP. **(c)** In group B (PEP-Cobb > RPP-Cobb), the Cobb angles in the HSP and PEP are significantly greater than the Cobb angle in the RPP. **(d)** and **(f)** In the total cohort and group B, the symmetrical index of paraspinal muscle activity at the apex vertebra (AVSI) is close to 1 in the RPP. The AVSI values in the HSP and PEP are significantly greater than 1 and significantly greater than the AVSI in the RPP. **(e)** In group A, the AVSI values in the HSP and RPP are close to 1 and do not significantly differ, while the AVSI in the PEP is significantly greater than 1 and significantly greater than the AVSI in the RPP. *p < 0.05, **p < 0.01, ***p < 0.001, compared with that in the RPP

All measurement data had excellent intra-examiner reproducibility (ICC 0.946–0.987) and inter-examiner reliability (ICC 0.903–0.962). The Cobb angles in the three postures are listed in Table 2. In the total cohort, the mean HSP-Cobb was significantly greater than the mean RPP-Cobb (*p* < 0.001), and the mean PEP-Cobb was not significantly different from the mean RPP-Cobb (*p* = 0.954). However, we observed that 13 patients had a significantly smaller mean PEP-Cobb than the mean RPP-Cobb (*p* = 0.007) (**Group A, PEP-Cobb < RPP-Cobb**) (Fig. 2), while 14 patients had a significantly greater mean PEP-Cobb than the mean RPP-Cobb (*p* < 0.001) (**Group B, PEP-Cobb > RPP-Cobb**) (Fig. 3). The Cobb angles of the three postures are shown in Fig. 4a–c. Among them, the numbers of exceeding a 5 degree difference in Cobb angle between positions are shown in Table 3. The subgroups showed no significant differences in demographic characteristics, except for the HSP-Cobb (Table 1).

**Table 2:** Cobb angles in the three test postures (mean ± SD)

Groups	HSP-Cobb (°)	RPP-Cobb (°)	p	PEP-Cobb (°)	p
Total cohort (*n* = 27)	30.59 ± 11.69	20.15 ± 10.12	0.000*	21.36 ± 11.13	0.954
Group A (PEP-Cobb < RPP-Cobb) (*n* = 13)	25.68 ± 8.64	20.13 ± 8.76	0.001*	16.18 ± 6.26	0.007*
Group B (PEP-Cobb > RPP-Cobb) (*n* = 14)	35.14 ± 12.57	20.17 ± 11.57	0.000*	26.16 ± 12.64	0.000*

HSP-Cobb: Cobb angle in the habitual standing position; RPP-Cobb: Cobb angle in the relaxed prone position; PEP-Cobb: Cobb angle in the prone extension position; Group A (PEP-Cobb < RPP-Cobb): patients in whom the PEP-Cobb is smaller than or equal to the RPP-Cobb; Group B (PEP-Cobb > RPP-Cobb): patients in whom the PEP-Cobb is greater than the RPP-Cobb. *p < 0.05, compared with RPP-Cobb

**Table 3:** The number of exceeding a 5 degree difference in Cobb angle between positions

	HSP-Cobb - RPP-Cobb>5° (n)	PEP-Cobb - RPP-Cobb>5° (n)	RPP-Cobb - PEP-Cobb ≥ 5° (n)
Total cohort (*n* = 27)	21	8	5
Group A (PEP-Cobb < RPP-Cobb) (*n* = 13)	7	0	5
Group B (PEP-Cobb > RPP-Cobb) (*n* = 14)	14	8	0

HSP-Cobb: Cobb angle in the habitual standing position; RPP-Cobb: Cobb angle in the relaxed prone position; PEP-Cobb: Cobb angle in the prone extension position; Group A (PEP-Cobb < RPP-Cobb): patients in whom the PEP-Cobb is smaller than or equal to the RPP-Cobb; Group B (PEP-Cobb > RPP-Cobb): patients in whom the PEP-Cobb is greater than the RPP-Cobb

## Surface electromyography results

### Asymmetry of the paraspinal muscle activity

The SI values of sEMG activities are listed in Table 4. In the total cohort, the SI of sEMG activity at the AV and LEV levels in the HSP were significantly greater than 1 (*p* = 0.006, *p* = 0.002, respectively) and the SI values at the UEV, AV, and LEV levels in the PEP were significantly greater than 1 (*p* = 0.012, *p* < 0.001, *p* = 0.001, respectively); however, the SI values at the three levels in the RPP were close to 1 (*p* = 0.471, *p* = 0.981, *p* = 0.130, respectively). In group A, the SI values at the three levels in the PEP were significantly larger than 1 (*p* = 0.016, *p* = 0.001, *p* = 0.046, respectively), and the SI value at the LEV level in the HSP was significantly larger than 1 (*p* = 0.023), while the SI values at the three levels in the RPP were close to 1 (*p* = 0.701, *p* = 0.753, *p* = 0.075, respectively). In group B, the SI values at the AV and LEV levels were significantly greater than 1 in the HSP (*p* = 0.016, *p* = 0.019, respectively) and PEP (*p* = 0.002, *p* = 0.004, respectively), while the SI values at the three levels were close to 1 in the RPP (*p* = 0.433, *p* = 0.925, *p* = 0.638, respectively).

**Table 4:** Symmetrical index of surface electromyographic activity in the three postures

Postures	Levels	Total cohort (*n* = 27)	p	Group A (PEP-Cobb < RPP-Cobb) (*n* = 13)	p	Group B (PEP-Cobb > RPP-Cobb) (*n* = 14)	P
HSP	UEV	1.09(0.92, 1.92)	0.058	1.03 (0.81, 1.61)	0.422	1.21 (0.97, 2.01)	0.064
	AV	1.24 (0.91, 2.50)	0.006*	1.05 (0.89, 1.67)	0.221	1.67 (0.98, 2.67)	0.016*
	LEV	1.25 (1.01, 2.05)	0.002*	1.13 (1.00, 1.58)	0.023*	1.45 (0.97, 3.25)	0.019*
RPP	UEV	0.89 (0.74, 1.17)	0.471	0.86 (0.75, 1.20)	0.701	0.92 (0.67, 1.15)	0.433
	AV	1.03(0.81, 1.15)	0.981	1.04 (0.81, 1.20)	0.753	0.94 (0.74, 1.28)	0.925
	LEV	1.07 (0.92, 1.57)	0.130	1.12 (0.96, 1.39)	0.075	0.96 (0.76, 1.91)	0.638
PEP	UEV	1.23 (0.99, 1.47)	0.012*	1.24 (1.13, 1.51)	0.016*	1.15 (0.90, 1.34)	0.272
	AV	1.55 (1.24, 1.93)	0.000*	1.30 (1.19,1.82)	0.001*	1.57 (1.24, 3.03)	0.002*
	LEV	1.23 (1.02, 1.69)	0.001*	1.15 (0.99, 1.64)	0.046*	1.34 (1.10, 2.35)	0.004*

Group A (PEP-Cobb < RPP-Cobb): patients in whom the Cobb angle in the prone extension position (PEP-Cobb) is smaller than or equal to the Cobb in the relaxed prone position (RPP-Cobb); Group B (PEP-Cobb > RPP-Cobb): patients in whom the PEP-Cobb is greater than the RPP-Cobb; HSP: habitual standing position; RPP: relaxed prone position; PEP: prone extension position; UEV: upper end vertebra; AV: apex vertebra; LEV: lower end vertebra; * *p* < 0.05, compared with 1

## Comparison of the SI between postures

In the total cohort, the SI values at the AV level (AVSI) significantly differed between the HSP, RPP, and PEP (*p* < 0.001); however, the SI values at the UEV and LEV levels in the three postures did not significantly differ (*p* = 0.093, *p* = 0.104, respectively). The AVSI were significantly larger in the HSP and PEP than the RPP (*p* = 0.043, *p* < 0.001, respectively) (Fig. 4d).

In group A, the SI values at the UEV and LEV levels in the three postures did not significantly differ (*p* = 0.232, *p* = 0.926, respectively); nevertheless, the AVSI in the three postures were significantly different (*p* = 0.037). Furthermore, the AVSI in the PEP were significantly larger than that in the RPP (*p* = 0.032); however, there was no significant difference in the AVSI between the HSP and RPP (*p* = 0.980) (Fig. 4e).

In group B, the SI values at the UEV in the three postures did not significantly differ (*p* = 0.145). However, the SI values at the LEV and the AVSI in the three postures were significantly different (*p* = 0.030, *p* = 0.004, respectively). Furthermore, the SI value at the LEV level in the HSP was significantly greater than that in the RPP (*p* = 0.042), and the AVSI were significantly greater in the HSP and the PEP than in the RPP (*p* = 0.042, *p* = 0.004, respectively) (Fig. 4f).

## Correlation between the AVSI and the Cobb angles

Because the AVSI in the PEP had the most significant difference among the three levels of the scoliotic curve, the AVSI in the PEP were further analyzed. In the total cohort and group B, the AVSI did not correlate with the Cobb angles in the three postures (Table 5). In group A, the AVSI showed a moderately significant positive correlation with the PEP-Cobb (*r* = 0.682, *p* = 0.01).

**Table 5:** Correlation between the AVSI in the PEP and the Cobb angles

	HSP-Cobb (r, p)	RPP-Cobb (r, p)	PEP-Cobb (r, p)
Total cohort (*n* = 27)	(0.317, 0.107)	(0.310, 0.115)	(0.347, 0,076)
Group A (PEP-Cobb < RPP-Cobb) (*n* = 13)	(0.527, 0.064)	(0.484, 0.094)	(0.682, 0.010)*
Group B (PEP-Cobb > RPP-Cobb) (*n* = 14)	(0.305, 0.288)	(0.219, 0.452)	(0.262, 0.365)

HSP-Cobb: Cobb angle in the habitual standing position; RPP-Cobb: Cobb angle in the relaxed prone position; PEP-Cobb: Cobb angle in the prone extension position; Group A (PEP-Cobb < RPP-Cobb): patients in whom the PEP-Cobb is smaller than or equal to the RPP-Cobb; Group B (PEP-Cobb > RPP-Cobb): patients in whom the PEP-Cobb is greater than the RPP-Cobb

## Discussion

## Changes of the Cobb angles and SI in the three postures in the total cohort

In the total cohort, the mean HSP-Cobb was significantly greater than the mean RPP-Cobb, which indicates that the gravitational load imposed on the upright spine is likely to be a contributory factor to the development and progression of AIS, and that the strength of the erector spinae muscles may be related to this process. The plausibility of this relationship may be explained by recognizing that the erector spinae muscles are antigravity muscle, which　contribute to the dorsal extension of the spine and also provide some lateral flexional force [27] We identified that the SI values of paraspinal muscle activity at the AV and LEV levels were significantly greater than 1 in the HSP. These findings do not support the previously reported findings suggesting that asymmetrical paraspinal muscle activities cause scoliosis, but may be a secondary result of increased Cobb angle. Therefore, the enhanced sEMG activities on the convex side may indicate that the erector spinae muscles are trying to stabilize the scoliotic spine.

In the total cohort, the mean PEP-Cobb did not significantly differ to the mean RPP-Cobb, while the erector spinae muscles had significant asymmetrical sEMG activities at the three levels (UEV, AV, and LEV) from RPP to PEP. A preliminary interpretation of this finding may suggest that asymmetrical sEMG activities of the erector spinae muscles may not impact the Cobb angle change. But in the process of x-ray measurement, we found 13 patients had smaller or unchanged Cobb angles, while 14 patients had greater Cobb angles from RPP to PEP. Therefore, the asymmetrical sEMG activities of the erector spinae muscles may be associated with Cobb angle change in some patients, in opposite direcions.

## Changes of the Cobb angles and SI in the three postures in the subgroups

For patients in group A, their PEP-Cobb was decreased or unchanged compared with their RPP-Cobb, and the AVSI of sEMG activity in the PEP showed significant asymmetry (AVSI > 1) and was significantly greater than that in the RPP. This suggests that in the PEP without axial load (body gravity) imposed on the spine, erector spinae muscle activity on the convex side may be used to lift the trunk and offset lateral bending moments produced by muscle activity on the concave side. Increased muscle activity on the convex side may produce more reverse lateral bending moments, which may result in a decrease in the Cobb angle. These results suggest that the asymmetrical muscle activity in group A may be an adaptive muscle response that attempts to correct scoliosis.

However, for patients in group B, their PEP-Cobb was greater than their RPP-Cobb, and the AVSI of sEMG activity in the PEP was also significantly greater than 1 and greater than that in the RPP. This seems to be the opposite of the findings in group A, and this difference may be due to the biomechanical and surrounding tissue structural differences in scoliosis compared with the normal spine [34]. Although the demographic characteristics of the two subgroups were not significantly different, the mean HSP-Cobb in group B was significantly larger than that in group A (Table 1). Due to the larger HSP-Cobb in group B, these patients may have had more severe changes in the morphological geometry of the erector spinae muscles, disc tilting, and connective tissue contracture, and had a greater deviation of the apex vertebra from the midline. Therefore, the lateral bending moment arm on the concave side may have been larger in group B than group (A) Additionally, due to the rotation and displacement of the vertebral bodies in group B, the erector spinae muscles on the convex side sometimes even protruded into the concave side, resulting in an increase in the torque on the concave side [35]. As a result, the PEP-Cobb was larger than the RPP-Cobb in group (B) These results suggest that with the progression of AIS, the role of the paraspinal muscles may change and become unfavorable to the stability of the scoliotic spine, such as group B.

## Correlation between the AVSI and the Cobb Angle

We found that the AVSI of the paraspinal muscles in the total cohort was significantly greater than 1 in the HSP and PEP, which was consistent with other studies [16, 20, 36, 37]. Asymmetrical sEMG activity of the paraspinal muscles has been observed in patients with AIS. The SI of the paraspinal muscle activity in the total cohort and two subgroups at the three levels was close to 1 in the RPP. Thus, it seems that asymmetrical paraspinal muscle activity only occurs in certain scenarios under muscular load. Additionally, the AVSI of the paraspinal muscle activity in the PEP had no correlation with the Cobb angle in the total cohort and group B. Only in group A, the AVSI in the PEP showed a moderately positive correlation with the PEP-Cobb demonstrating that the larger the RPP-Cobb angle, the greater AVSI was needed to maintain the corresponding larger PEP-Cobb angle. These findings including total cohort, group B and group A are not fully consistent with those of previous studies [24, 38, 39] which report the higher the angle of curvature, the greater the erector spinae muscle activity on the convex side. It is possible that with the progression of scoliosis, the role of the paraspinal muscles becomes unfavorable to the stability of the scoliotic spine, and the stabilizing role is mainly played by passive structures (vertebrae, discs, and ligaments ) [40], causing a relative decrease in the erector spinae muscle activity on the convex side. Since the AVSI changes (from symmetrical to asymmetrical) correspond to two different Cobb angle changes and the AVSI in the PEP had no correlation with the Cobb angle in the total cohort and group B, the increased sEMG activity of the paraspinal muscles on the convex is more likely to be an attempt to stabilize and correct the scoliotic spine in the early stage of AIS.

## Rehabilitation therapy

Previous review articles have reported that exercise therapy has potential benefits in treating the physiological and psychological aspects of patients with AIS [41, 42]. However, it remains controversial whether it is best to exercise the convex or concave paraspinal muscles. Some authors have argued that a reduction in the bioelectric activity on the concave side shows that the muscle is weaker than on the convex side, which leads to postural deficits. Thus, strengthening of the paraspinal muscles on the concave side might improve scoliosis [43, 44]. However, Chwała et al. [45] thought that muscle strengthening on the concave side could potentially exacerbate tension on the concave side of the curved spine, which is known as the “bowstring effect”. Our results also support the view that strengthening the paraspinal muscles on the convex side might be beneficial in patients with mild AIS [26, 45, 46], such as those in group (A) Increased muscle activity on the convex side shows that the muscle is trying to stabilizing the spine in the HSP, and more muscle activity is needed to maintain spine stability. However, symmetrical training might be harmful to patients with moderate and severe AIS, such as those in group (B) For patients with AIS, it might be more beneficial to perform asymmetrical training that aims to strengthen the weakened muscle on the convex side and stretch the tight muscle on the concave side to correct trunk stability and improve muscle imbalance [46]. This causes eccentric contraction of the shortened muscles and concentric contraction of the elongated muscles to obtain symmetrical sEMG activity. If exercise causes the lateral bending moment produced by the paraspinal muscles on the convex side to be equal to or greater than the lateral bending moment produced by gravity and the paraspinal muscles on the concave side, this may stop or correct the progression of scoliosis. Exercise therapy is based on Neuromuscular control re-training and muscle strengthening [47]. The effectiveness of asymmetrical training should be further explored in future studies.

Transcutaneous electrical muscle stimulation was reported to be a reasonable alternative to arrest the progression of AIS [48]. Electrostimulation on the right side of the spine resulted in a left convex in the rabbits [49]. But others reported electrical stimulation was ineffective in preventing curve progression of AIS [50, 51]. Our results also agree with that increased muscle activity on the convex may not the contributory factor to the development of scoliosis.

## Strengths and limitations

The present study has some strengths. Previous studies have used sEMG to assess the paraspinal muscle performance of patients with AIS during static and dynamic tasks [16, 25] and used standing whole-spine radiographs to determine the severity of scoliosis. However, few studies have focused on the effect of sEMG activity on the scoliotic Cobb angle in the coronal plane during these tasks. It is known that the erector spinae muscles are activated less in the RPP [24, 45] and more in the PEP [29]. In the present study, we used sEMG to observe the asymmetrical activities of the paraspinal muscles and used radiography to evaluate the Cobb angle in the coronal plane in different postures to investigate the effect of asymmetrical erector spinae muscle activity on the Cobb angle in the coronal plane in AIS. Our investigation showed that the role of the paraspinal muscles may change with the progression of scoliosis.

The present study also has some limitations. First, this was a preliminary study of the role of the paraspinal muscles in the development of AIS, while the effect of asymmetrical muscles activities on vertebral rotation was not analyzed. Second, the multifidus, which also play an important role in stabilizing the spine and controlling the lordosis [28], were not included. Third, X-ray examinations and sEMG measurements are not conducted simultaneously, but at intervals of 3–5 days. Fourth, the sEMG results may have been affected by unintentional activity at the time of measurement. Fifth, the small sample size, inclusion of only patients with single-curve scoliosis, and lack of comparative analysis with a normal age-matched group may bring some bias in reflecting the clinical outcomes. Patients with double-curve scoliosis and a control group will be included in future studies.

## Conclusion

Asymmetrical sEMG activity of the paraspinal muscles exists in the specific task in AIS patients, especially at AV level. The Cobb angle and SI change, as the position varies. Gravitational load imposed on the upright spine is likely to be a contributory factor to the development and progression of AIS. Asymmetrical sEMG activity of the paraspinal muscles may be not an inherent feature of AIS patients, but is evident in the challenging tasks. The potential significance of asymmetric paraspinal muscle activity need to be explored in further research.

## Data Availability

The data used to support the findings of this study are available from the corresponding author upon request.

## Abbreviations

sEMG: surface electromyography
AIS: adolescent idiopathic scoliosis
HSP: habitual standing position
RPP: relaxed prone position
PEP: prone extension position
RMS: root mean square
SI: symmetrical index
UEV: upper end vertebra
AV: apex vertebra
LEV: lower end vertebra
AVSI: the SI of sEMG activity at the apex vertebra level
RPP-Cobb: Cobb angle in the relaxed prone position
PEP-Cobb: Cobb angle in the prone extension position
HSP-Cobb: Cobb angle in the habitual standing position
ICC: the intraclass correlation coefficient
